# Regulatory effects of intermittent noxious stimulation on spinal cord injury-sensitive microRNAs and their presumptive targets following spinal cord contusion

**DOI:** 10.3389/fncir.2014.00117

**Published:** 2014-09-18

**Authors:** Eric R. Strickland, Sarah A. Woller, Sandra M. Garraway, Michelle A. Hook, James W. Grau, Rajesh C. Miranda

**Affiliations:** ^1^Department of Neuroscience and Experimental Therapeutics, College of Medicine, Texas A&M Health Science CenterBryan, TX, USA; ^2^Department of Psychology, Texas A&M University, College StationTX, USA

**Keywords:** spinal cord injury, microRNA, uncontrollable nociception, BDNF, IGF

## Abstract

Uncontrollable nociceptive stimulation adversely affects recovery in spinally contused rats. Spinal cord injury (SCI) results in altered microRNA (miRNA) expression both at, and distal to the lesion site. We hypothesized that uncontrollable nociception further influences SCI-sensitive miRNAs and associated gene targets, potentially explaining the progression of maladaptive plasticity. Our data validated previously described sensitivity of miRNAs to SCI alone. Moreover, following SCI, intermittent noxious stimulation decreased expression of miR124 in dorsal spinal cord 24 h after stimulation and increased expression of miR129-2 in dorsal, and miR1 in ventral spinal cord at 7 days. We also found that brain-derived neurotrophic factor (BDNF) mRNA expression was significantly down-regulated 1 day after SCI alone, and significantly more so, after SCI followed by tailshock. Insulin-like growth factor-1 (IGF-1) mRNA expression was significantly increased at both 1 and 7 days post-SCI, and significantly more so, 7 days post-SCI with shock. MiR1 expression was positively and significantly correlated with IGF-1, but not BDNF mRNA expression. Further, stepwise linear regression analysis indicated that a significant proportion of the changes in BDNF and IGF-1 mRNA expression were explained by variance in two groups of miRNAs, implying co-regulation. Collectively, these data show that uncontrollable nociception which activates sensorimotor circuits distal to the injury site, influences SCI-miRNAs and target mRNAs within the lesion site. SCI-sensitive miRNAs may well mediate adverse consequences of uncontrolled sensorimotor activation on functional recovery. However, their sensitivity to distal sensory input also implicates these miRNAs as candidate targets for the management of SCI and neuropathic pain.

## INTRODUCTION

Significant attention has been given to investigating the central molecular changes that modulate locomotor recovery and pain following spinal cord injury (SCI; Boyce and Mendell, 2014; Lerch et al., 2014; Schwab and Strittmatter, 2014; Silva et al., 2014). However, surprisingly, there is relatively little information on the role of afferent peripheral signals in the development of SCI-related pain or attenuation of locomotor function. Given that SCI rarely occurs in isolation, but is often accompanied by peripheral tissue damage and trauma, examining the effects of afferent nociceptive signaling seems warranted. This is further underscored by previous research in our laboratory suggesting that functional recovery after SCI is attenuated by peripheral nociceptive stimulation applied below the level of the spinal injury (Grau et al., 2004). Intermittent noxious stimulation decreased the recovery of locomotor function, delayed reinstatement of bladder function, led to greater mortality and spasticity, and increased tissue loss at the injury site in adult rats (Grau et al., 2004). Further, we found that nociceptive stimulation rapidly resulted in decreased brain-derived neurotrophic factor (BDNF) – tropomyosin-receptor kinase B signaling (Gomez-Pinilla et al., 2007; Garraway et al., 2011). This signaling pathway has been shown to play a key role in modulating neuronal plasticity, promoting axonal regeneration, and improving functional recovery following SCI (Xu et al., 1995; Patterson et al., 1996; McTigue et al., 1998; Kerr et al., 1999; Garraway et al., 2003; Boyce et al., 2007; Huie et al., 2012b).

The present study focused on identifying intracellular regulatory molecules that, when activated by nociceptive input, might influence neurotrophic signaling pathways and functional recovery. Specifically, we hypothesized that miRNAs may be sensitive to nociceptive stimulation, and activate potentially maladaptive gene networks to further undermine recovery after SCI. We, and others, have previously shown that SCI leads to significant alterations in miRNA expression (Liu et al., 2009; Nakanishi et al., 2010; Strickland et al., 2011). Our previous study showed that miR1, miR124, and miR129 were significantly down-regulated following a spinal cord contusion, while miR146a and miR21 were transiently induced (Strickland et al., 2011), and that these miRNAs were sensitive to opioid analgesics like morphine (Strickland et al., 2014). These miRNAs play important roles in regulating critical gene networks that have been implicated in the development of hypersensitivity and may contribute to impaired functional recovery. For example, though the expression of miR124 is often associated with early neuronal differentiation (Visvanathan et al., 2007), and neurite outgrowth (Yu et al., 2008a), miR124 is also important for promoting neuronal survival in the adult (Doeppner et al., 2013). Moreover, miR124 may play an important role in neuropathic pain by inhibiting neuro-inflammation (Ponomarev et al., 2011; Kynast et al., 2013) and inflammatory hyperalgesia (Willemen et al., 2012) in the adult. Similarly, miR129-2 promotes cell cycle arrest, and dysregulation of miR129-2 results in neuronal death (Di Giovanni et al., 2003; Byrnes et al., 2007; Herrup and Yang, 2007; Wu et al., 2010). Interestingly, miR1, a member of the miR1/miR206 family has been shown to regulate important growth-promoting neurotrophic factors like BDNF (Lewis et al., 2003; Lee et al., 2012). Modulation of miR1 activity may play a significant role in regulating BDNF signaling after SCI and, importantly for this study, in mediating the effects of intermittent noxious stimulation on recovery after SCI. To assess this possibility, the current study investigated the relationship between SCI-sensitive miRNA expression and intermittent noxious stimulation in tissue collected 1 h, 24 h, and 7 days after stimulation. We also assessed the relationship between the expression of miRNAs sensitive to intermittent noxious stimulation and that of their mRNA targets.

## MATERIALS AND METHODS

### SUBJECTS

The subjects were male Sprague–Dawley rats (*Rattus norvegicus*) obtained from Harlan (Houston, TX, USA). The rats were approximately 90–110 days old (350–400 g), were individually housed in Plexiglas bins [45.7 (length) × 23.5 (width) × 20.3 (height) cm] with food and water continuously available, and were maintained on a 12-h light/dark cycle. All behavioral testing was performed during the light cycle. To facilitate access to the food and water, extra bedding was added to the bins after surgery and long mouse sipper tubes were used so that the rats could reach the water without rearing. All of the experiments were reviewed and approved by the Institutional Animal Care and Use Committee at Texas A&M University and all NIH guidelines for the care and use of animal subjects were followed.

### SURGERY

Subjects were anesthetized with isoflurane (5%, gas). Once a stable level of anesthesia was achieved, the concentration of isoflurane was lowered to 2–3%. An area extending approximately 4.5 cm above and below the injury site was shaved and disinfected with iodine, and a 7.0 cm incision was made over the spinal cord. Next, two incisions were made on either side of the vertebral column (approximately 4–5 mm depth), extending about 3 cm rostral and caudal to the T12 segment. The dorsal spinous processes at T12 vertebral level were removed (laminectomy), and the spinal tissue exposed. The dura remained intact. For the contusion injury, the vertebral column was fixed within the MASCIS device (Gruner, 1992; Constantini and Young, 1994), and a moderate injury was produced by allowing the 10 g impactor (outfitted with a 2.5 mm tip) to drop 12.5 mm. Sham controls received a laminectomy, but the cord was not contused with the MASCIS device. Following surgery, the wound was closed with Michel clips. T12 vertebral level contusion models have been routinely used by members of our group to define spinal cord learning circuits and molecular mechanisms involved with recovery of function (Ferguson et al., 2008; Brown et al., 2011; Hook et al., 2011). Lesions at this level result in well-defined and replicable sensory-motor deficits, and we therefore chose to utilize contusion at this level to also examine changes in miRNA expression.

To help prevent infection, subjects were treated with 100,000 units/kg Pfizerpen (penicillin G potassium) immediately after surgery and again 2 days later. For the first 24 h after surgery, rats were placed in a recovery room maintained at 26.6°C. To compensate for fluid loss, subjects were given 2.5 ml of saline after surgery. Bladders were manually expressed twice daily (morning and evening) until the animals had empty bladders for three consecutive days, at the times of expression.

### UNCONTROLLABLE INTERMITTENT NOXIOUS STIMULATION

Intermittent shock was applied 24 h after surgery while subjects were loosely restrained in Plexiglas tubes as previously described (Crown et al., 2002; Grau et al., 2004). Electrical stimulation was applied through cutaneous electrodes coated with electrode gel (Harvard Apparatus, Holliston, MA, USA) and attached 2 cm from the tip of the tail with Orthaleic tape. Leads from the electrodes were attached to a shock generator (BRS/LVE, Model SG-903, Laurel, MD, USA), and intermittent constant current 1.5 mA, electrical stimulation was applied through the electrodes. Rats treated with uncontrollable intermittent stimulation received 180, 80-ms tail shocks on a variable time schedule with a mean inter-stimulus interval of 2 s (range 0.2–3.8 s). Unshocked subjects (contused and sham operated) were placed in the restraining tubes for the same amount of time as shock subjects. The unshocked subjects had the tail electrodes attached, but did not receive the electrical stimuli. The number of subjects within each experimental condition is given **Table 1**.

**Table 1:** Number of subjects (*n*) per condition.

Treatment	1 h	1 day	7 days
Sham operated	4	4 (d and v)	4 (d and v)
Contused-unshocked	6	6 (d and v)	6 (d and v)
Contused-shocked	6	6 (d and v)	6 (d and v)

*Tissue collected at 1 and 7 days postsurgery was subdivided into dorsal (d) and ventral (v) portions and separately analyzed.*

### RNA EXTRACTION AND qRT-PCR

Animals [SCI/unshocked (SCI_unshock_) or SCI/shock-exposed (SCI_shock_)] were sacrificed at 1 h, 1 day, or 7 days following exposure to intermittent shock or control treatment (i.e., 25 h, 48 h, and 8 days following SCI). All subjects were deeply anesthetized with pentobarbital (50 mg/kg), and 1 cm of spinal cord around the lesion epicenter was rapidly removed. To determine the spatial (dorsal–ventral) changes in the expression of miRNAs and genes of interest, the spinal cord tissue was further subdivided to yield dorsal and ventral regions in the 1 and 7 days after treatment groups. The cord was processed for the extraction of total RNA (RNeasy Mini Kit; Qiagen, Valencia, CA, USA) and subsequently quantified using a NanoDrop 2000 Spectrophotometer (Thermo Scientific; Wilmington, DE, USA), and stored at -80°C.

MiRNA expression data was measured with quantitative reverse transcription (qRT)-PCR for miRNAs, based on the protocol of the miRCURY^TM^ LNA microRNA Universal RT-PCR system (EXIQON; Woburn, MA, USA). RNA samples were converted to cDNA, and qRT-PCR was performed using a 7900HT Fast Real-Time PCR System (Applied Biosystems, Foster City, CA, USA). Validated commercially available forward and reverse primers (EXIQON; **Table 2**) for hsa-miR124, hsa-miR1, hsa-miR21, hsa-miR129-2, and hsa-miR146a were used for PCR amplification, and real time data was normalized to U6 small nuclear RNA (U6_SNR_). Similarly, mRNA expression of BDNF and IGF-1 was measured using qRT-PCR for mRNAs, based on the protocol for PerfeCTa®; SYBR®; Green SuperMix with ROX^TM^ (Quanta Biosciences; Gaithersburg, MD, USA). RNA samples were converted to cDNA using qScript^TM^ cDNA SuperMix (Quanta Biosciences; Gaithersburg, MD, USA), and qRT-PCR was performed using a 7900HT Fast Real-Time PCR System (Applied Biosystems, Foster City, CA, USA). Forward and reverse primers (Integrated DNA Technologies; Coralville, IA, USA) for BDNF and IGF-1 were used for PCR amplification (**Table 2**), and real time data was normalized using glyceraldehyde 3-phosphate dehydrogenase (GAPDH). Three technical replicates were averaged per sample. Selection criteria for non-commercially provided mRNA primer pairs were the identification of a single amplicon and efficiencies based on serial cDNA dilution according to our previously published protocols (Camarillo and Miranda, 2008). Efficiency for mRNA primers was 100.4% for BDNF, 99.71% for IGF-1 and 100.06% for GAPDH. Normalization controls were utilized as reported previously (Strickland et al., 2011, 2014), and there were no statistical differences in the expression of either U6_SNR_ or GAPDH with treatment condition or spinal cord region. Relative miRNA and mRNA expression was determined by calculating the mean difference between cycle threshold of either the miRNA from the U6_SNR_ normalization control, or the BDNF/IGF-1 mRNA from the GAPDH normalization control for each sample (Δ cycle threshold (ΔCT)) within each sample group (samples with same miRNA ID, time, and condition parameters) and expressed as -ΔCT for relative change in expression. Sample means that were greater than ±2 SDs from the mean ΔCT were considered outliers and removed from the analysis. As indicated in **Table 1**, the experimental design yielded 80 samples and for each, seven miRNAs were assessed, yielding a total of 560 data points. Our criteria identified just 30 data points for exclusion (approximately 5.4%), and no more than one data point in any individual experimental group was excluded. Fold change in miRNA/mRNA expression was determined by calculating the difference between the mean ΔCT for the sham control and individual sample ΔCTs of sham, SCI/unshocked (SCI_unshock_), and SCI/shock-exposed (SCI_shock_) sample groups at the same time point (-ΔΔCT), and expressed as mean fold-change (2^-ΔΔCT^; Livak and Schmittgen, 2001).

**Table 2:** Primer sequences.

mRNA Primers	Forward	Reverse
BDNF	TGGACATATCCATGACCAGAAA	CACAATTAAAGCAGCATGCAAT
IGF-1	CCGCTGAAGCCTACAAAGTC	GGGAGGCTCCTCCTACATTC
GAPDH	AGTATGTCGTGGAGTCTACTG	TGGCAGCACCAGTGGATGCAG

**miRNA Primers/cat#**	**Target Sequence**	**Sequence reference**

hsa-miR-1/#204344	UGGAAUGUAAAGAAGUAUGUAU	MIMAT0000416
hsa-miR-21-5p/#204230	UAGCUUAUCAGACUGAUGUUGA	MIMAT0000076
hsa-miR-124-3p/#204319	UAAGGCACGCGGUGAAUGCC	MIMAT0000422
hsa-miR-129-2-3p/# 204026	AAGCCCUUACCCCAAAAAGCAU	MIMAT0004605
hsa-miR-146a-5p/# 204688	UGAGAACUGAAUUCCAUGGGUU	MIMAT0000449
U6 snRNA/# 203907		

### DATA ANALYSIS

All data were analyzed using SPSS software version 18 (SPSS; Chicago, IL, USA). MicroRNA expression, verified by qRT-PCR, was analyzed by multivariate analysis of variance (ANOVA) using Pillai’s trace statistic, and further analyzed using *post hoc* univariate ANOVA and Fisher’s least significant difference (LSD) test. Other data were analyzed using ANOVAs followed by *post hoc* f-LSD using planned comparisons to limit the number of *post hoc* comparisons. In all cases, the *a priori α* value was set at 0.05. Data were expressed as mean ± SEM, as indicated in the figure legends.

Correlations between expression of miRNAs, and between miRNA and either BDNF or IGF-1 mRNA expression, were determined by Pearson’s product–moment correlation using –ΔCT values of either the miRNAs or BDNF/IGF-1 as separate independent variables. The *a priori α* value was set at 0.05, and data were expressed as the mean difference in cycle threshold change of either each miRNA relative to the cycle threshold of U6 controls (-ΔCT = CT_U6_ - CT_miRNA_), or BDNF or IGF-1 expression relative to the cycle threshold of GAPDH controls (-ΔCT = CT_GAPDH_ –CT_mRNA_). Additionally, stepwise linear regression analyses were performed on Pearson’s correlation data between miRNA and either BDNF or IGF-1 mRNA expression to determine which miRNAs contributed to the significant correlations. BDNF or IGF-1 mRNA expression was the dependent variable, while miRNA expression was the independent variable, and the *a priori α* value was set at 0.05 for model significance.

## RESULTS

### QUANTIFICATION OF SHOCK-INDUCED CHANGES IN miRNA EXPRESSION

#### Contused rats exhibited increased miR21 and miR146a expression 1 h after shock treatment

**FIGURE 1:**
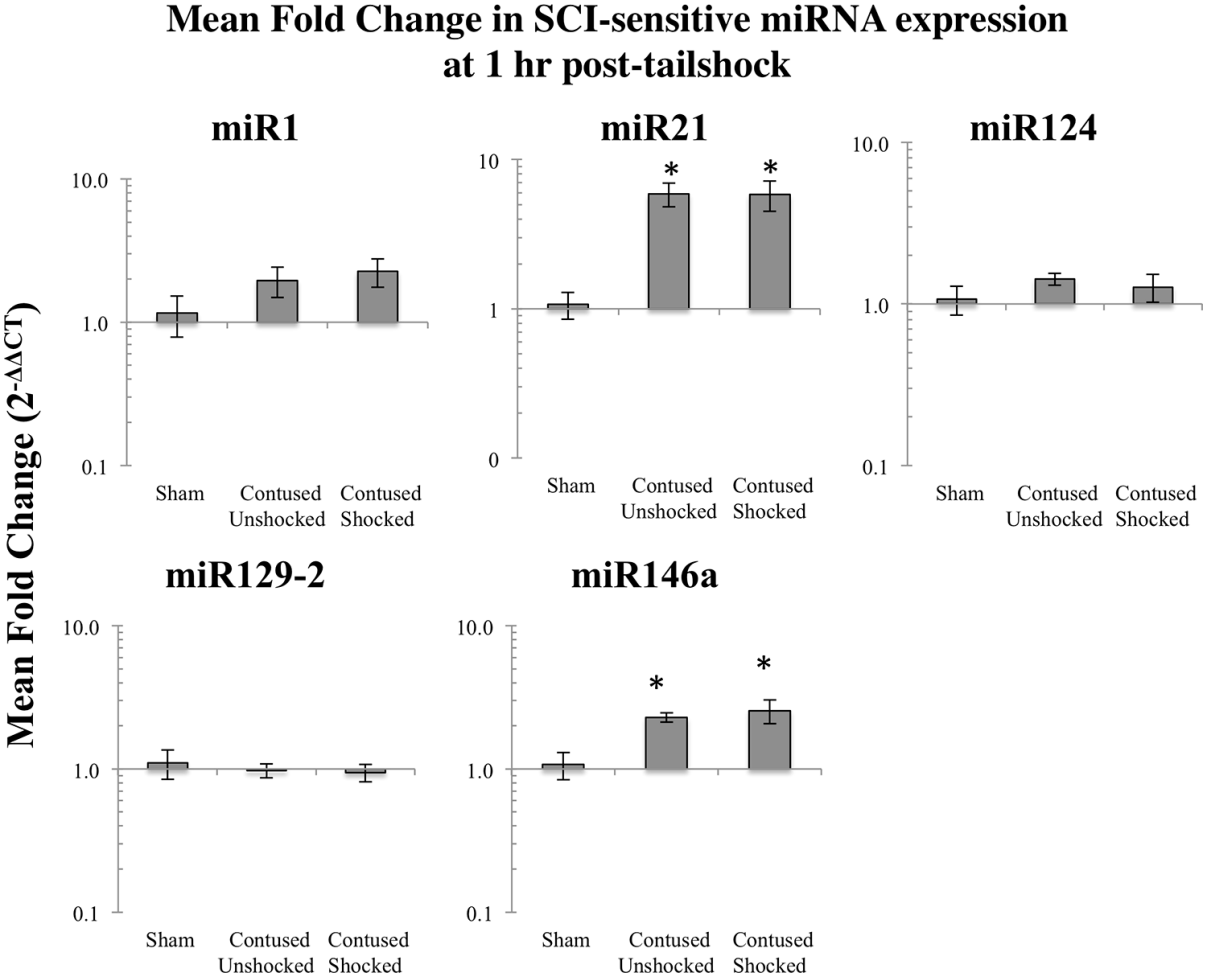
**Bar graphs depicting qRT-PCR analysis of miRNA expression of miR1, miR21, miR124, miR129-2, and miR146a at the lesion site for sham animals and after unshocked or shock treatment in contused animals at 1 h following tailshock treatment.** The *x*-axis denotes treatment condition, and the *y*-axis denotes the log of mean fold expression change of miRNA. Data are normalized relative to U6 small nuclear RNA internal gene expression and expressed as the mean of all fold changes of individual samples within a treatment group relative to the mean of the treatment group, with asterisks indicating significance (LSD) compared with sham controls; *p* values are as indicated in the text. The error bars indicate the standard error of the mean (SEM).

We previously reported that miR1, miR21, miR124, miR129-2, and miR146a were significantly affected by a spinal cord contusion (Strickland et al., 2011). To determine whether uncontrollable intermittent noxious stimulation affects these SCI-sensitive miRNAs, their expression was determined by qRT-PCR in sham controls and in contused animals following either no shock exposure (SCI_unshock_) or uncontrollable intermittent tailshock (SCI_shock_). Initially, we analyzed miRNA expression within the whole spinal cord segment (combined dorsal and ventral spinal cord) at the lesion site at 1 h following intermittent noxious stimulation (25 h after contusion surgery). Both miR21 [*F*_(2,13)_ = 15.4, *p* < 0.0003] and miR146a [*F*_(2,13)_ = 6.2, *p* < 0.01] were significantly increased following SCI (all *post hoc* comparisons relative to sham control, *p* < 0.02), and exposure to intermittent tail shock did not result in further alterations of miRNA expression (**Figure 1**). MiR1, miR124, and miR129-2 were not significantly altered at the lesion site, either by contusion or by intermittent tail-shock at 1 h post-stimulation.

#### A contusion injury down-regulated expression of miR1, miR124, mi129-2, and up-regulated miR21, 1–7 days after treatment

We microdissected the 1 cm of spinal cord tissue bracketing the injury segment to yield dorsal/sensory and ventral/motor regions in the 1 and 7 days treatment groups. Microdissection of the cord provides additional information on the spatial (dorsal–ventral) and functional relevance (sensory-motor) of changes in SCI-sensitive miRNA expression. We hypothesized that SCI-induced expression changes of some trauma-sensitive miRNAs would exhibit spatial and functional specificity, and that uncontrollable nociception might exacerbate these changes. miRNA expression in contused rats was significantly altered by exposure to shock and the pattern of expression depended upon both time and region (dorsal versus ventral). Preliminary analyses using a multivariate ANOVA of qRT-PCR data confirmed that there was a significant main effect of time [Pillai’s Trace Statistic, *F*_(5,33)_ = 20.89; *p* < 0.001], treatment [*F*_(10,68)_ = 6.44; *p* < 0.001], and spinal region (dorsal/ventral) [*F*_(5,33)_ = 8.31; *p* < 0.001], as well as a three-way statistically significant interaction between time, treatment, and spinal region [*F*_(10,68)_ = 2.59; *p* < 0.01]. *Post hoc* univariate ANOVAs indicated a main effect of time on miR1, miR21, miR124, and miR146a, a main effect of treatment on miR1, miR21, miR124, and miR129-2, and a main effect of spinal region on miR1 and miR146a (all *F*s > 9.14, *p* < 0.005). There was also a significant interaction effect of time and treatment on miR124 expression, and of time, treatment, and spinal region on miR1, miR129-2, and miR146a (all *F*s > 3.68, *p* < 0.05). An effect of time emerged because expression generally declined across days. miR1 and miR146a exhibited an overall difference across spinal regions because expression was somewhat less in the ventral portion.

**FIGURE 2:**
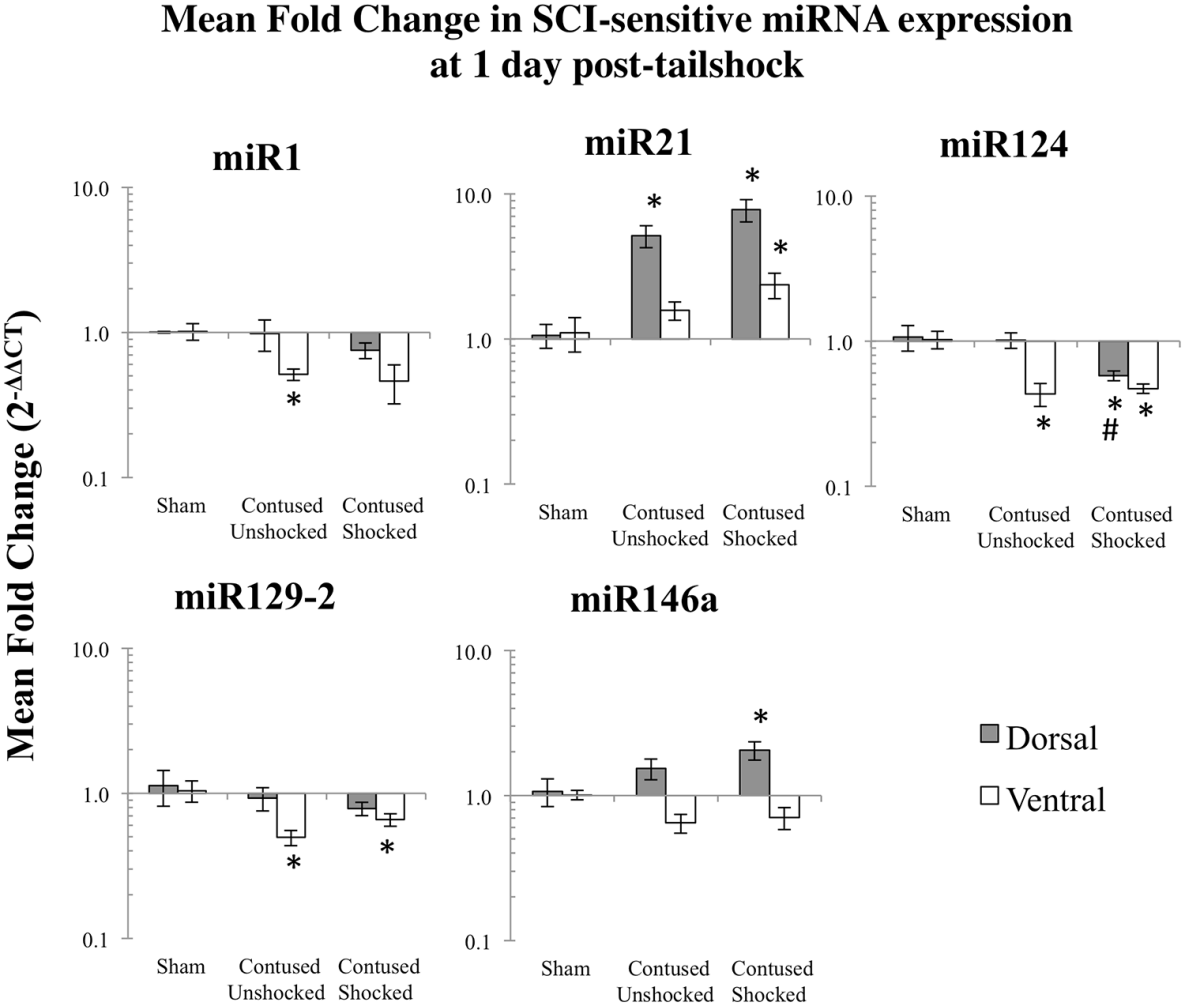
**Bar graphs depicting qRT-PCR analysis of miRNA expression of miR1, miR21, miR124, miR129-2, and miR146a at the lesion site for sham animals and after unshocked or shock treatment in contused animals at 1 day following tailshock treatment.** The *x*-axis denotes treatment condition, and the *y*-axis denotes the log of mean fold expression change of miRNA. Data are normalized relative to U6 small nuclear RNA internal gene expression and expressed as the mean of all fold changes of individual samples within a treatment group relative to the mean of the treatment group (+SEM), with asterisks indicating significance (LSD) compared with sham controls and hash tags indicating significance (LSD) compared with contused unshocked controls; *p* values are as indicated in the text.

**FIGURE 3:**
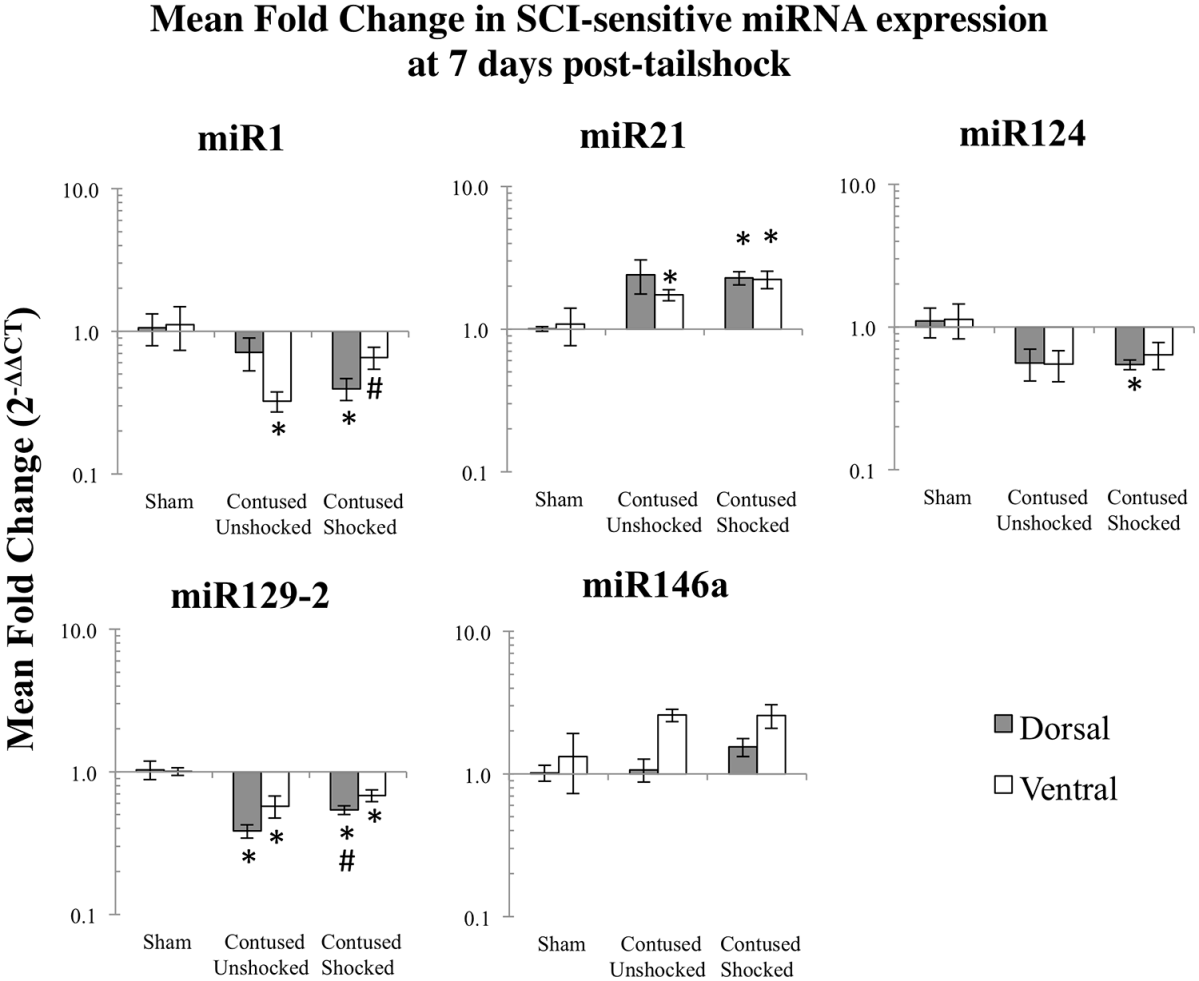
**Bar graphs depicting qRT-PCR analysis of miRNA expression of miR1, miR21, miR124, miR129-2, and miR146a at the lesion site for sham animals and after unshocked or shock treatment in contused animals at 7 days following tailshock treatment.** The *x*-axis denotes treatment condition, and the *y*-axis denotes the log of mean fold expression change of miRNA. Data are normalized relative to U6 small nuclear RNA internal gene expression and expressed as the mean of all fold changes of individual samples within a treatment group relative to the mean of the treatment group (+SEM), with asterisks indicating significance (LSD) compared with sham controls and hash tags indicating significance (LSD) compared with contused unshocked controls; *p* values are as indicated in the text.

*Post hoc* LSD *t*-tests were used to further analyze the effect of treatment. We found that miR1, miR124, and miR129-2 expression was significantly decreased following spinal cord trauma, irrespective of exposure to uncontrollable intermittent tailshock (*p*_miR1_ < 0.001, *p*_miR124_ < 0.001, and *p*_miR129-2_ < 0.001; **Figures 2** and **3**). In contrast, miR21 expression was up-regulated in contused subjects (*p*_miR21_ < 0.001), and there was no change in the expression of miR146a.

#### Intermittent tailshock produces a selective increase in mR1/miR129-2 and a decrease in miR124 in contused rats

The effect of shock treatment also depended upon region and time (**Figures 2** and **3**). The SCI_shock_ rats alone exhibited a significant down-regulation of miR1 and miR124 in the dorsal region of the spinal cord at 7 days posttreatment. *Post hoc* planned comparisons also indicated that the contusion injury *per se* decreased the expression of miR1 in the ventral/motor tissue, but this effect was significantly attenuated by uncontrollable shock; expression of miR1 was increased in the SCI_shock_ group compared with SCI_unshock_ treatment. Similarly, there was a significant increase in the expression of miR129-2 in dorsal/sensory tissue at 7 days following SCI_shock_ treatment relative to SCI_unshock_ treatment (*p* < 0.05; **Figure 3**), and a significant decrease in the expression of miR124 in dorsal/sensory tissue at 1 day following SCI_shock_ relative to SCI_unshock_ treatment (*p* < 0.01; **Figure 2**).

#### Correlation analyses indicated significant relationships between expression changes within distinct groups of SCI-sensitive miRNAs

**FIGURE 4:**
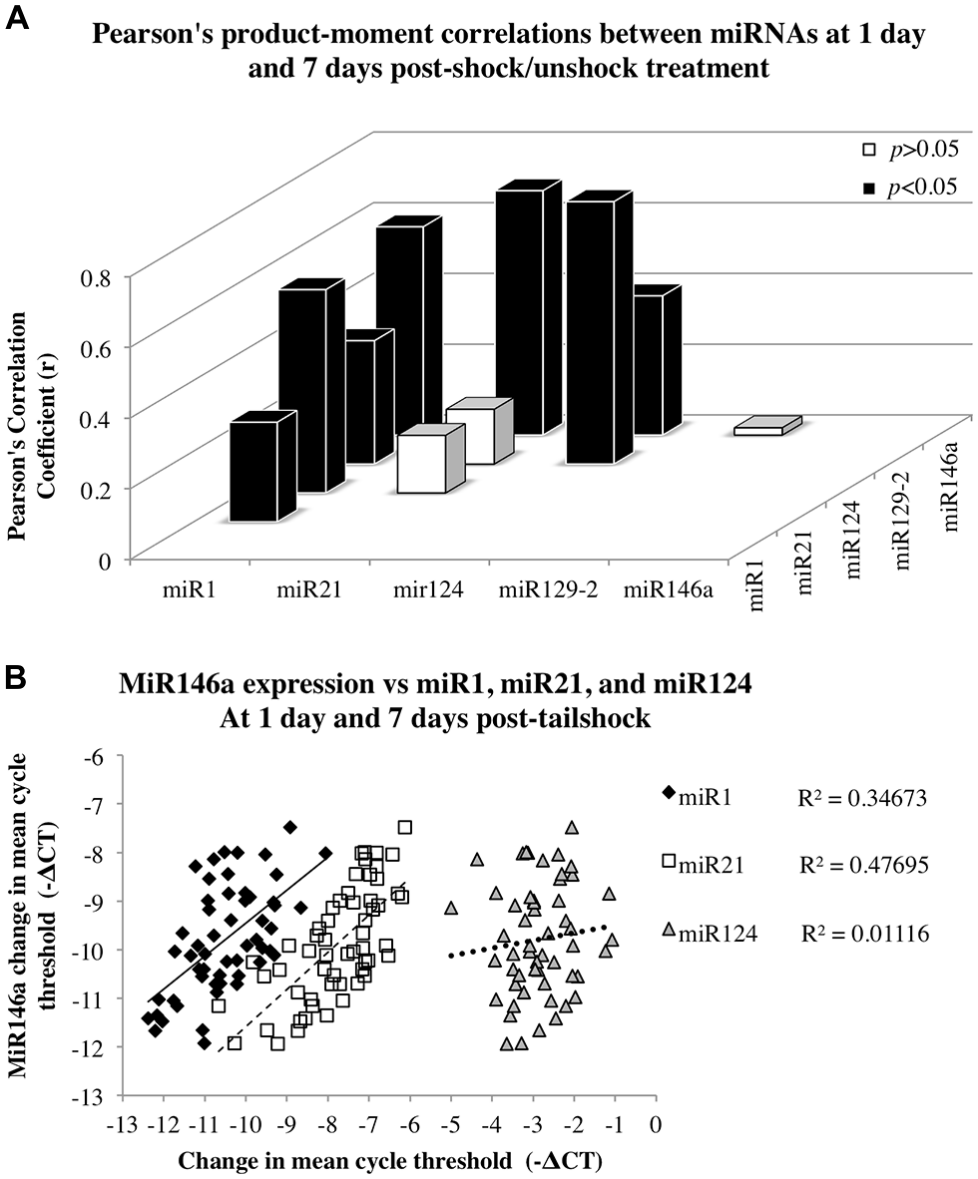
**Correlation analyses to assess the relationship between miR146a miRNA expression and expression of miR1, miR21, and miR124 following SCI.** Data for dorsal and ventral spinal cord and for day 1 and 7 post-shock were combined for analysis. **(A)** The *x*-axis and *z*-axis represent the miRNAs of interest, and the *y*-axis depicts the magnitude of the Pearson’s product–moment correlation coefficient, *r*, between the corresponding miRNAs indicated on the *x*- and *z*-axes. Pearson’s correlations indicated seven statistically significant relationships (*p* < 0.05; black bars) and three non-significant relationships (*p*> 0.05; white bars). **(B)** The *x*-axis represents the mean difference in cycle threshold change (-ΔCT) between miRNA expression at both dorsal and ventral spinal cord and at 1 and 7 days following tailshock treatment and U6 controls, and the y-axis depicts the mean difference in cycle threshold change (-ΔCT) between miR146a expression in dorsal and ventral spinal cord and at both 1 and 7 days following tailshock treatment and U6 controls. Pearson’s correlations indicated a significant correlation between miR1 (black diamonds), miR21 (white squares), and miR124 (gray triangles), and miR146a (Pearson’s *r* = 0.59, *P* < 0.001, Pearson’s *r* = 0.69, *P* < 0.001, and Pearson’s *r* = 0.39, *P* < 0.005, respectively). Data are represented as the mean change in cycle threshold of miRNA expression relative to U6 controls (-ΔCT = CT_U6_ - CT_miRNA_).

Given miRNA dysregulation following both contusion injury and uncontrollable nociception, we hypothesized that statistical relationships might exist between expression changes of different SCI-sensitive miRNAs, indicative of possible co-regulation. Pearson’s product–moment correlations indicated three groups of significant correlations between miRNAs (**Figures 4A,B**; **Table 3**). In combined analysis of data obtained from both dorsal and ventral spinal cord, and at both 1 and 7 days, there were significant correlations between miR1 and miR21, miR124, miR129-2, and miR146a (**Figure 4A**), between miR124 and miR1, miR129-2, and miR146a, and between miR146a and miR1, miR21, and miR124 (for all Pearson’s *r*s, *p* < 0.05, see **Table 3**; **Figure 4B**).

**Table 3:** Pearson’s product–moment correlations for miRNA expression.

	miR1	miR21	miR124	miR129-2	miR146a
**miR1**
Pearson correlation		0.282*	0.575**	0.349**	0.589**
Sig. (two-tailed)		*0.034*	*0.001*	*0.01*	*0.001*
*N*		*57*	*54*	*54*	*54*
**miR21**
Pearson correlation			0.163	-0.155	0.691**
Sig. (two-tailed)			*0.227*	*0.255*	*0.001*
*N*			*57*	*56*	*57*
**miR124**
Pearson correlation				0.742**	0.394**
Sig. (two-tailed)				*0.001*	*0.003*
*N*				*57*	*56*
**miR129-2**
Pearson correlation					0.021
Sig. (2-tailed)					*0.876*
*N*					*56*

Analyses were based on combined data for both dorsal and ventral spinal cord and at 1 and 7 days following tailshock treatment. For each correlation between miRNAs given by row and column heading, the Pearson’s Correlation Coefficient, *r*, the *p* value based on Student’s two-tailed *t*-test, and the sample number, *N*, are listed in order from top to bottom, respectively. Three main clusters of miRNAs correlate significantly together: miR1 correlates with miR21, miR124, miR129-2, and miR146a, miR124 correlates with miR1, miR129-2, and miR146a, and miR146a correlates with miR1, miR21, and miR124.

*Correlation is significant at the 0.05 level (two-tailed).

**Correlation is significant at the 0.01 level (two-tailed).

### INTERMITTENT SHOCK SENSITIVITY OF miRNA TARGET GENES IN CONTUSED RATS

#### Contused rats exhibited a decrease in IGF-1 expression at 1 h

**FIGURE 5:**
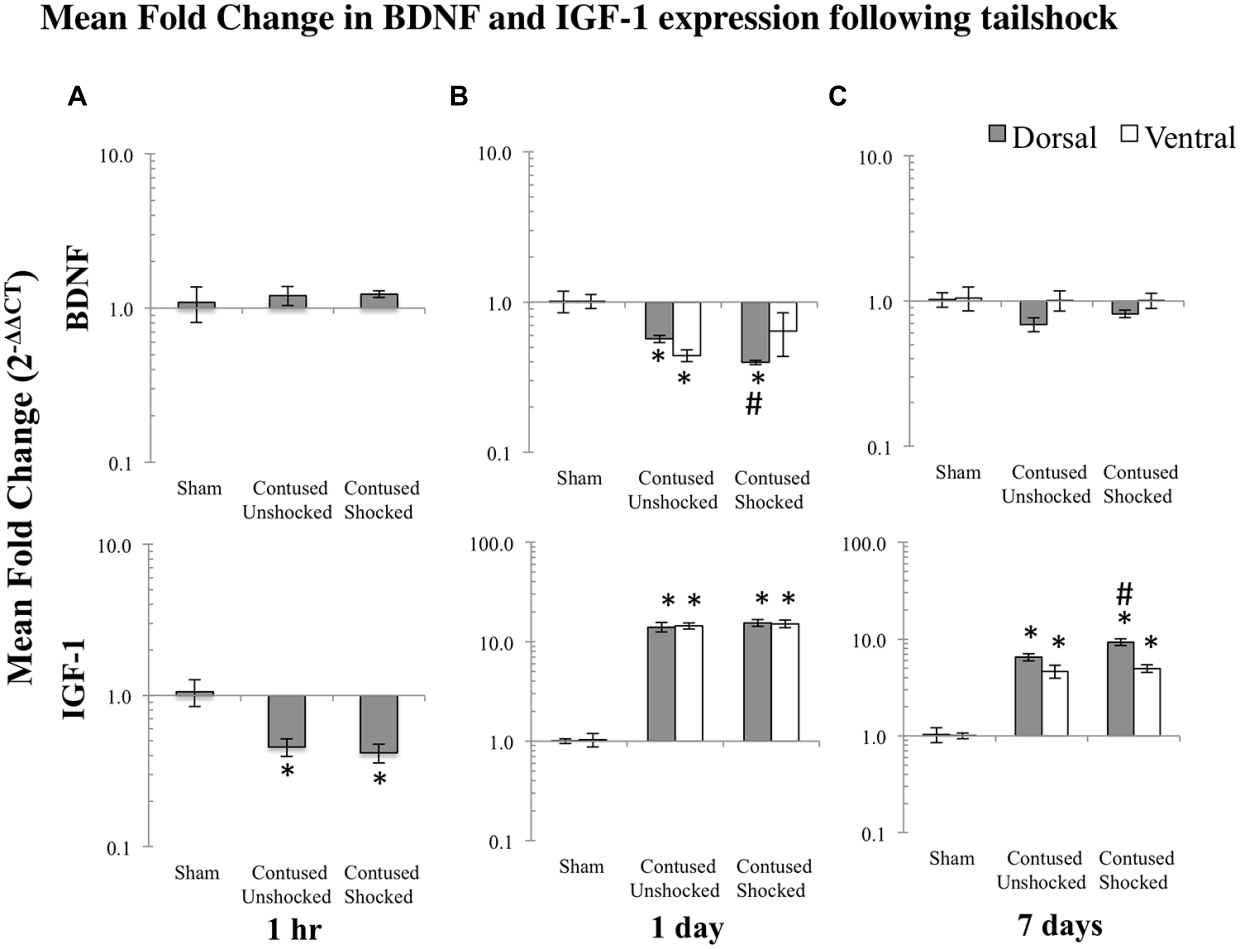
**Bar graphs depict qRT-PCR analysis of BDNF and IGF-1 mRNA expression at the lesion site following shock treatment in contused animals, compared to sham lesioned controls and non-shocked (unshock), lesioned animals at 1 h (A), 1 day (B), or 7 days (C) following tailshock treatment.** The *x*-axis denotes treatment condition, and the *y*-axis denotes the log of mean fold expression change of mRNA. Data were normalized relative to GAPDH mRNA and expressed as the mean of fold changes of individual samples within a treatment group relative to the mean of the treatment group (+EM). As indicated in the text, there were significant main effects of time and spinal cord region (dorsal versus ventral) on mRNA expression. Within each time group, asterisks indicate significance (LSD) compared with time-group specific sham controls and hash tags indicate significance (LSD) compared with contused unshocked controls; *p* values are as indicated in the text.

As miR1 is further modified by uncontrollable intermittent tailshock following contusion, we assessed the extent to which changes in miR1 expression corresponded to modulation of potential neurotrophin and growth factor mRNA targets. Based on prior work (Lewis et al., 2003; Yu et al., 2008b; Lee et al., 2012; Li et al., 2012), we assayed BDNF and IGF-1 mRNA expression by qRT-PCR in sham controls and in response to either no shock exposure or uncontrollable intermittent tailshock following injury. As with the miRNA expression analysis, we first analyzed mRNA expression in full segments (dorsal + ventral) at 1 h following treatment. Planned comparisons indicated that there was a significant decrease in expression of IGF-1 following the contusion injury, irrespective of uncontrollable intermittent tailshock (Student’s two-tailed *t*-test, *p*_unshock_ < 0.01 and *p*_shock_ < 0.005; **Figure 5A**).

#### A contusion injury down-regulated expression of BDNF and up-regulated IGF-1 mRNA expression

Subsequently, spatial expression (dorsal/sensory–ventral/motor) of BDNF and IGF-1 mRNA was assessed at 1 and 7 days following SCI_unshock_ or SCI_shock_ treatment. A multivariate ANOVA of the qRT-PCR data indicated a significant main effect of time [Pillai’s Trace Statistic, *F*_(2,51)_ = 5.20; *p* < 0.01], treatment [*F*_(4,104)_ = 11.66; *p* < 0.001], and spinal region (dorsal/ventral) [*F*_(2,51)_ = 17.97; *p* < 0.001], and a significant interaction effect between both time and treatment [*F*_(4,104)_ = 6.29; *p* < 0.001], and treatment and spinal region [*F*_(4,104)_ = 2.56; *p* < 0.05]. *Post hoc* univariate ANOVAs indicated a main effect of time on IGF-1 mRNA, a main effect of treatment on both BDNF and IGF-1 mRNAs, and a main effect of spinal region on BDNF mRNA (all *F*s > 9.49, *p* < 0.005). For IGF-1 and BDNF mRNA expression, the effect of treatment depended on time (both *F*s > 7.95; p < 0.001). In addition, for BDNF the effect of treatment depended upon spinal region as well as time (all *F*s > 4.03; *p* < 0.05).

*Post hoc* LSD *t*-tests indicated that BDNF mRNA was significantly decreased, while IGF-1 mRNA was significantly increased following spinal cord trauma, (*p* < 0.001; **Figures 5B,C**).

#### Intermittent tailshock reduces BDNF expression in the dorsal region a day after treatment and increases IGF-1 expression at 7 days following treatment

*Post hoc* planned comparisons indicated that there was a significant decrease in expression of BDNF in dorsal/sensory tissue of SCI_shock_ subjects relative to SCI_unshock_ at 1 day following tailshock (*p* < 0.05; **Figure 5B**). In contrast, shock treatment increased expression of IGF-1 mRNA in ventral/motor tissue at 7 days following SCI_unshock_ or SCI_shock_ treatment (*p* < 0.05; **Figure 5C**). *Post hoc* planned comparisons also indicated significant spatial changes (dorsal/sensory relative to ventral/motor) in mRNA expression, including increased relative dorsal/sensory expression of BDNF in sham, SCI_unshock_, and SCI_shock_ treated subjects at both 1 day (*p* < 0.01, *p* < 0.001, and *p* < 0.05, respectively) and 7 days following SCI_unshock_ or SCI_shock_ treatment (*p* < 0.01, *p* < 0.01, and *p* < 0.001, respectively), and increased expression of IGF-1 in SCI_shock_ subjects at 7 days following SCI_unshock_ or SCI_shock_ treatment (*p* < 0.005; **Figures 5B,C**).

#### Correlation analyses indicated that expression changes of multiple SCI-sensitive miRNAs were associated with changes in BDNF and IGF-1 expression

As BDNF and IGF-1 can be mRNA targets of miR1, we hypothesized that a statistical relationship would exist between expression changes in miR1 and that of BDNF and IGF-1. Pearson’s product–moment correlations (combining data for dorsal and ventral spinal cord and for day 1 and 7 post-shock) indicated two groups of significant correlations between SCI-sensitive miRNAs, BDNF, and IGF-1. There were significant correlations between BDNF and both miR21 and miR124, and between IGF-1 and miR1, miR124, and miR129-2 (**Table 4**). Additionally, BDNF and IGF-1 were also significantly, but inversely correlated with each other (Pearson’s *r* = -0.31, *p* < 0.05).

**Table 4:** Pearson’s product–moment correlations between miRNA expression and the expression of Igf1 and BDNF mRNA transcripts.

	miR1	miR21	miR124	miR129-2	miR146a
**BDNF mRNA**
Pearson correlation	0.069	0.332**	-0.289*	-0.164	0.154
Sig. (two-tailed)	*0.609*	*0.009*	*0.024*	*0.211*	*0.244*
*N*	*57*	*60*	*61*	*60*	*59*
**Igf1 mRNA**
Pearson correlation	0.402**	-0.235	0.469**	0.415**	0.173
Sig. (two-tailed)	*0.002*	*0.071*	*0.001*	*0.001*	*0.189*
*N*	*57*	*60*	*61*	*60*	*59*

Analysis was based on combined data for dorsal and ventral spinal cord and from 1 and 7 days following tailshock treatment. For each correlation between miRNAs given by row and column heading, the Pearson’s correlation coefficient, *r*, the *p* value based on Student’s two-tailed *t*-test, and the sample number, *N*, are listed in order from top to bottom, respectively.

*Correlation is significant at the 0.05 level (two-tailed).

**Correlation is significant at the 0.01 level (two-tailed).

We also used multivariate analyses to discover which miRNAs accounted for a significant proportion of the variance in IGF-1 and BDNF mRNA expression. For IGF-1, the analyses revealed that miR124, miR21, and miR146a (in that order) each accounted for an independent proportion of the variance (all *F*s > 6.36, p < 0.05), and together explained 42.3% of the variance. BDNF mRNA expression was largely explained by miR21 and miR124 expression (both *F*s > 7.57, *p* < 0.001), which together explained 24.8% of the variance. For both mRNAs, no other miRNA independently explained a significant proportion of variance.

## DISCUSSION

One of the current clinical challenges with mitigating the short- and long-term effects of spinal cord injury is managing the uncontrollable nociception that results from concomitant peripheral tissue damage. It is critical for future efforts to develop therapeutic strategies to attenuate glial activation, cell death, and sensitization of spinal neurons associated with intermittent noxious stimulation in order to inhibit maladaptive spinal plasticity, improve functional recovery, and suppress pain hypersensitivity following SCI with associated peripheral injuries (Liu et al., 1999; Frei et al., 2000; Beattie et al., 2002; Hains et al., 2003; Xu et al., 2004; Hains and Waxman, 2006; Lampert et al., 2006; Detloff et al., 2008; Kuzhandaivel et al., 2011). Uncontrollable nociception clearly inhibits functional recovery (Grau et al., 2004; Garraway et al., 2011; Huie et al., 2012a). It is possible that nociceptive stimuli may interfere with functional recovery in part, by directly altering biology within damaged tissue at the injury site. Specifically, it is possible that miRNAs, given their dysregulation following SCI (Liu et al., 2009; Nakanishi et al., 2010; Strickland et al., 2011, 2014; Bhalala et al., 2012) and ability to coordinate the expression of large gene networks, are involved in the modulation of nociceptive circuitry and prevention of functional recovery resulting from uncontrollable nociception. Therapeutic manipulation of these miRNAs could alleviate the maladaptive effects of noxious stimulation in both the acute and chronic phases of SCI by suppressing activation of glia-mediated inflammation and inhibiting the synaptic remodeling of nociceptive circuitry that results in pain hypersensitivity, increased spasticity, and exacerbation of behavioral deficits.

The current study investigated the regulation of SCI-sensitive miRNAs at the site of an injury in rats administered uncontrollable shock (SCI_shock_) or nothing (SCI_unshock_) 1 h, 1 day, or 7 days after shock treatment. To examine the effect of a contusion injury *per se*, we included a sham-operated unshocked control as a comparison group. We found that the contusion injury increased expression of miR21 and miR146a at 1 h relative to the sham control. In the unshocked rats, miR21 remained elevated in the dorsal (at 1 day) and ventral (at 7 days) regions. In contused shocked rats, miR21 remained elevated in both the dorsal and ventral region across days (1 and 7). The change in miR146 appeared, in contrast, to fade over days, with the only significant increase observed in the dorsal region of shocked rats at 1 day. At 1 day, the contusion injury down-regulated miR1, miR124, and miR129-2 in the ventral region of unshocked subjects. miR1 remained down-regulated in the ventral region at 7 days and miR129-2 was down-regulated in both the dorsal and ventral regions. Shocked rats also showed a down-regulation of miR124 (dorsal and ventral) and miR129-2 (ventral) at 1 day. At 7 days, only shocked-contused rats exhibited a significant down-regulation of miR1 and miR124 in the dorsal region, whereas miR129-2 was down-regulated in both regions. This overall pattern replicates key components of our earlier study (Strickland et al., 2011), which showed that a contusion injury down-regulates miR1, miR129, and miR124, and up-regulates miR21 and miR146a.

When we examined the effect of shock treatment on miRNA expression in contused rats, we found that shock significantly decreased expression of miR124 in dorsal/sensory tissue at 1 day. At 7 days, shock treatment significantly increased the expression of miR1 (ventral region) and miR129-2 (dorsal region), relative to injured, unshocked controls. This is the first evidence that miRNA networks at the site of a contusion injury are affected by uncontrollable stimulation to distal spinal segments. Systemic factors like inflammatory stimuli (Huie et al., 2012a) and the activation of glia (Vichaya et al., 2009) may mediate the effects of distal uncontrollable stimulation on miRNA expression. However, the direct involvement of intra-spinal circuitry cannot be eliminated. Previous tract-tracing studies in the rat have described intra-spinal proprioceptive circuits between the sacro-caudal spinal cord that exerts sensorimotor control over the tail and more rostral spinal levels including thoracic segments (Masson et al., 1991). Moreover, spinal cord trauma has also been previously shown to result in extensive rewiring of intra-spinal circuitry (Bareyre et al., 2004) in the rat. While rewired circuitry matures over a period of several weeks following injury (Bareyre et al., 2004), a combination of undamaged and early rewiring intra-spinal circuits may constitute a mechanism whereby distal nociceptive stimuli regulate miRNA expression at the injury site. Interestingly, shock treatment shifted the regional distribution miRNA expression in contused rats, leading to decreased expression of miR124 in the dorsal region and at 7 days, a region-specific up-regulation of miR1 (ventral) and miR129-2 (dorsal) relative to unshocked animals. In addition, differences in expression of miR124 were accounted for by variation in the expression of miR1, miR129-2, and miR146a, suggesting that these miRNAs may be co-regulated. Collectively, these data suggest that uncontrollable nociception modulates SCI-sensitive miRNA networks in a spatially specific manner.

We observed that although there was initially significant down-regulation of IGF-1 following contusion, uncontrolled intermittent shock did not immediately result in additional suppression of Igf-1 at the 1-h time point. In contrast, IGF-1 was significantly increased in response to prior contusion at both 1 and 7 days following shock/unshock exposure, in both dorsal/sensory and ventral/motor tissue. Interestingly uncontrollable intermittent tailshock induced additional significant up-regulation only after a temporal delay, i.e, at 7 days after treatment, and only in the dorsal (i.e., sensory) region of the spinal cord. These data suggest that the IGF-1 response of the dorsal spinal cord is more responsive to nociceptive influences compared to the ventral spinal cord.

Consistent with our previous findings (Garraway et al., 2011), we found that BDNF expression was significantly down-regulated at 1 day, as a function of SCI, but that uncontrollable intermittent tail-shock resulted in additional and significant BDNF suppression, within the dorsal/sensory region of the spinal cord. A step-wise linear regression analysis revealed that variation in miR124, miR21, and miR146 accounted for a significant proportion of the variation in IGF-1 mRNA expression, and that miR21 and miR124 accounted for variation in BDNF mRNA expression, suggesting that both BDNF and IGF-1 regulation involves a network of miRNAs. Alternatively, BDNF and IGF-1 may also influence miRNA expression and the potential role of their down-stream signaling cascades in influencing the expression of mR21 and miR124 need further investigation. The correlated expression of miRNA and trophic factor transcripts may also point to the existence of positive feed-forward regulatory pathways. For example, recent research has shown that another member of the neurotrophin family, nerve growth factor (NGF) induces the expression of miR21, and miR21 in turn promoted NGF-induced neuronal differentiation (Montalban et al., 2014). Following SCI such correlated trophic factor-miRNA networks may be important for promoting neuroprotection.

Surprisingly, though miR1 and IGF-1 were both responsive to uncontrollable nociception variation in miR1 did not account for a significant proportion of the variation in IGF-1 (or BDNF). This surprising finding perhaps reflects physiological differences between dorsal-sensory and ventral-motor spinal cord and their association with nociceptive stimulation. At day 7 following nociceptive/shock stimulation, miR1 expression in dorsal spinal cord in animals that had only received SCI were not different from controls, whereas IGF-1 mRNA expression was significantly increased. Uncontrollable nociception resulted in a significant decrease in miR1 while further increasing IGF-1 mRNA. Therefore, in the dorsal, sensory spinal cord, nociceptive stimulation at least resulted in a prototypic inverse relationship between miRNA and target gene. Since the dorsal spinal cord is likely to be the primary recipient of nociceptive input, the inverse relationship between miR1 and IGF-1 may represent a direct sensory modulation of SCI and SCI-sensitive miRNA function. However, in ventral spinal cord, at 7 days following uncontrollable shock, animals that received SCI alone exhibited a significant decrease in miR1 and an increase in IGF1. While uncontrollable nociceptive stimulation in SCI animals attenuated this decrease in miR1 observed following SCI alone, effectively resulting in increased miRNA expression, the expression of IGF-1 remained unchanged from the shock-only condition. These data suggest a dissociation between miRNA and target gene networks following SCI and intermittent noxious stimulation, as we have previously observed in other models of neural damage (Pappalardo-Carter et al., 2013). While such dissociation may be simply the result of miRNA and mRNAs being expressed in distinct and non-overlapping cell cohorts, this dissociation in ventral spinal cord may be a secondary consequence of activating the dorsal spinal cord. It is also intriguing to hypothesize that this dissociation may indicate the emergence of intervening, compensatory biological mechanisms, including perhaps shifts in miRNA function from translation repression to transcription activation (Place et al., 2008; Liu et al., 2013).

Uncontrollable nociception may have significant adverse consequences for recovery of function, that may be mediated by SCI-sensitive miRNAs. These miRNAs have also been shown to be activated and to influence cellular responses to neuropathic pain. For example, consistent with our own observation showing delayed reduction in miR1, others have reported that peripherally applied nociceptive and inflammatory stimuli also result in long term reduction of miR1 expression in dorsal root ganglia (Kusuda et al., 2011). An accumulating literature suggests that several SCI-sensitive miRNAs control inflammatory and other biological functions that are pertinent to nociception. For example, miR124 suppresses activation of resting microglia and macrophages prior to injury, and both miR21 and miR146a have been shown to negatively regulate astrocyte activation following SCI (Ponomarev et al., 2011; Bhalala et al., 2012; Iyer et al., 2012; Willemen et al., 2012; Kynast et al., 2013). MiR124 is an especially important candidate, as it is involved in the regulation of both BDNF and IGF-1, is sensitive to uncontrollable intermittent tailshock, and has been shown to inhibit nociceptive behavior associated with neuropathic pain (Kynast et al., 2013). While these miRNA changes were assessed within the spinal cord, and presumably directly influenced by activation of neural nociceptive pathways, the role of intermediate physiological and cellular activators cannot be ignored. For example, changes in heart rate, blood pressure, or immune system activation in response to nociceptive stimuli may mediate changes in miRNA expression. In this context, it is important to note that miR1 is also highly expressed in the vascular system and increases the barrier capacity of endothelial cells (Wang et al., 2013). One prediction is that the persistent decrease in miR1 expression following SCI may lead to increased vascular permeability and consequently be permissive for leukocyte infiltration. In the early stages of injury, the suppression of miR1 may well have a protective effect, since the predicted increase in leukocyte infiltration may limit tissue damage and promote clearance of debris (Peruzzotti-Jametti et al., 2014). Uncontrollable nociceptive stimuli may be predicted to prevent or delay that effect.

SCI and nociception-sensitive target genes like IGF-1 also have important consequences for functional recovery. IGF-1 promotes both oligodendrocyte survival after SCI upon up-regulation through leukemia inhibitory factor-mediated activation of microglia (Kerr and Patterson, 2005; Mekhail et al., 2012), and reduces blood–brain barrier permeability and edema through attenuation of nitric oxide synthase up-regulation upon its topical application prior to and following SCI (Sharma et al., 1997; Nyberg and Sharma, 2002). IGF may have general neuroprotective value in a variety of disease models. For example, recent research has shown that IGF-1 protects against ischemic stroke (Selvamani et al., 2012). Intriguingly, in the above report, antisense-mediated suppression of miR1 had the same neuroprotective effect. These data suggest that the decrease in miR1 and increase in IGF-1 mRNA following SCI may represent a neuroprotective adaptation. Conversely, uncontrollable nociceptive stimulation effectively increases miR1 at least in dorsal spinal cord, and may therefore adversely influence functional recovery.

Collectively, these data suggest that SCI-sensitive miRNAs constitute an important component of a response to uncontrollable nociception from peripheral injury. While it remains to be ascertained, these miRNAs and their targets may well engage direct astrocyte- and glial-mediated mechanisms as well as indirect inflammatory pathways. Consequently, these miRNAs may constitute therapeutic targets for attenuating neuropathic pain following SCI.

## Conflict of Interest Statement

The authors declare that the research was conducted in the absence of any commercial or financial relationships that could be construed as a potential conflict of interest.
